# Comparative evaluation of sputum microscopy and Xpert MTB/RIF assay for tuberculosis detection and rifampicin resistance surveillance in Biratnagar, Nepal

**DOI:** 10.1128/spectrum.02460-25

**Published:** 2026-03-11

**Authors:** Shristy Ojha, Akash Adhikari, Sujan Adhikari, Swagat Khanal, Chandan Sah Kalwar, Om Prakash Panta

**Affiliations:** 1 Department of Microbiology, Central Campus of Technology 128243 , Dharan, Nepal; 2 Institute of Agriculture and Animal Science, Paklihawa Campus 676862 , Bhairahawa, Nepal; 3 Faculty of Animal Science, Veterinary Science and Fisheries, Agriculture and Forestry University 580598 , Chitwan, Nepal; 4 Sam Higginbottom University of Agriculture, Technology and Sciences 75647 , Prayagraj, India; University at Albany, Albany, New York, USA

**Keywords:** tuberculosis, Xpert MTB/RIF, drug resistance, diagnostics, Nepal

## Abstract

Tuberculosis (TB) control in Nepal faces persistent challenges due to underdiagnosis and increasing drug resistance. This study assessed the diagnostic performance of the Xpert MTB/RIF assay compared with conventional smear microscopy and estimated the prevalence of rifampicin-resistant TB (RR-TB) in Biratnagar, a high-burden urban center. Between March and September 2023, 509 clinical specimens from TB suspects were analyzed by Ziehl-Neelsen (ZN) staining, fluorescence microscopy (FM), and Xpert MTB/RIF. Using Xpert as the operational reference, ZN and FM exhibited overall sensitivities of 55.9% (89/159) and 68.5% (109/159), respectively. In paired sputum-only analyses (*n* = 446; sputum Xpert positives = 145), ZN and FM sensitivities were 60.0% (87/145) and 70.3% (102/145), respectively; sputum specificity was 100% for both methods. Xpert detected 31.2% (159/509) positive cases—78.7% more than ZN and 45.9% more than FM. Rifampicin resistance was identified in 5.03% (8/159) of Xpert-positive samples and was strongly associated with prior treatment (*P* < 0.001); RR-TB occurred in 66.7% (6/9) of retreatment cases vs 1.3% (2/150) of new cases. Xpert increased detection in paucibacillary and extrapulmonary specimens. These findings support expanded use of rapid molecular testing, but results should be interpreted cautiously because culture/speciation was not performed.

**IMPORTANCE:**

The spread of drug-resistant tuberculosis (TB) is a growing public health threat in Nepal. Using a rapid molecular test in Biratnagar, we simultaneously diagnosed TB and identified rifampicin resistance. We found rifampicin-resistant TB in >5% of Xpert-positive patients, higher than national averages for new cases, and a markedly elevated risk among previously treated patients. Rapid detection of resistance permits earlier initiation of appropriate therapy and can reduce onward transmission.

## INTRODUCTION

Tuberculosis (TB) remains one of the top 10 causes of death worldwide (1). Although TB is both preventable and curable with current therapies, the disease nonetheless resulted in 10.8 million new cases and 1.25 million deaths worldwide in 2023. The number of TB case notifications has rebounded to 8.2 million, which is the highest since the World Health Organization (WHO) began monitoring. This reflects recovery in health systems rather than a reduction in transmission. The overall TB deaths declined slightly compared to 2022, but the disease still kills more people than any other single bacterial infection (2, 3). TB remains an enormous burden in Nepal, with the WHO estimating 68,000 new TB cases and 16,000 TB-related deaths in 2023 (4). National TB Prevalence Surveys, which showed that the true burden was 1.6 times higher than previously estimated, reveal a vast detection gap. There were 40,776 cases notified in the fiscal year 2023/2024, with a considerable number of cases going undiagnosed (5). This represents a notification gap of approximately 40%, where over 27,000 individuals with TB were likely missed by the health system in a single year. Additionally, TB control initiatives in numerous nations have been hampered by the recent COVID-19 pandemic. The reduced testing and treatment access contributed to a backlog of undiagnosed cases (6, 7), fueling community-level transmission.

The emergence of drug resistance poses a significant threat to TB control efforts (8, 9). Multidrug-resistant tuberculosis (MDR-TB) is defined as a disease caused by *Mycobacterium tuberculosis* strains that are resistant to at least isoniazid and rifampicin, the two most potent first-line anti-TB medications (10). Rifampicin resistance (RR-TB) is considered a strong proxy for MDR-TB as it serves as a primary indicator to initiate second-line therapy. Globally, approximately 400,000 individuals suffered from MDR or RR-TB in 2023, but only 44% were accurately diagnosed and treated (2), showcasing a concerning diagnostic and treatment gap. This gap is greatly responsible for the continued transmission of drug-resistant strains, which are much harder to treat. Furthermore, it contributes to excess morbidity and mortality, along with increased healthcare expenditure. RR-TB is one of the key facets jeopardizing the global antimicrobial resistance framework since it reduces the effectiveness of one of the few and potent anti-TB drugs available (11). It also reflects the wider scenario of more and more bacterial pathogens evading standard treatment therapy. Enhanced surveillance of rifampicin-resistant TB is, therefore, essential to tailor appropriate treatment regimens and meet international TB control targets (12).

In many low- and middle-income countries, sputum smear microscopy has been the primary method for diagnosing tuberculosis for many years. It is both inexpensive and easy to perform, along with providing quick results (13). However, this method has a crucial drawback. It has highly variable sensitivity, typically ranging from 20% to 60%. This is especially challenging for patients with a low bacillary load, which includes children, HIV-positive individuals, and extrapulmonary TB cases (14).

A further critical limitation of sputum smear microscopy is its inability to differentiate between *Mycobacterium tuberculosis* complex (MTBC) and nontuberculous mycobacteria (NTM). As both are acid-fast bacilli, they are indistinguishable by microscopic examination, leading to misdiagnosis and inappropriate therapy. The global prevalence of NTM disease is rising, with particularly high rates reported in East Asia (15, 16). The misdiagnosis of NTM pulmonary disease as TB can lead to the prescription of ineffective and potentially toxic anti-TB drugs, while delaying appropriate therapy. Molecular diagnostics, such as the Xpert MTB/RIF assay, which specifically detect MTBC DNA, overcome this limitation and are therefore essential for accurate diagnosis (17).

To overcome these issues, the WHO approved Xpert MTB/RIF assay for use in diagnosing TB in children and HIV patients in 2010 (18). The Xpert MTB/RIF assay is a sophisticated test and is classified as a nucleic acid amplification test, utilizinga cartridge-based system. It was designed to rapidly detect the DNA of *Mycobacterium tuberculosis* and, critically, to simultaneously detect rifampicin resistance by identifying mutations in the *rpoB* gene (19). This fully automated system provides results in under 2 h with high sensitivity and requires substantially less technical proficiency than conventional culture, making it highly suitable for resource-limited settings. Starting in 2011, a national effort was made toward TB detection that included expanding the GeneXpert network with more than 50 diagnostic centers. However, several critical operational bottlenecks are hindering the network’s widespread effectiveness (20, 21).

There is a growing threat of drug resistance in the management of TB cases. The surveillance of rifampicin resistance is critical to guide appropriate treatment and improve patient outcomes. This study aimed to compare the bacteriological yield of sputum microscopy (Ziehl-Neelsen [ZN] and fluorescence microscopy [FM]) with Xpert MTB/RIF and estimate the prevalence of rifampicin resistance among TB suspects presenting to a DOTS-Plus Centre in Biratnagar, Nepal. This study will help in formulating effective control measures for TB, upgrading diagnostic methods, and bolstering the national efforts towards TB elimination.

## MATERIALS AND METHODS

### Study design and population

A cross-sectional study was conducted between March and September 2023 at the National Anti-Tuberculosis Association (NATA) DOTS Plus Centre in Biratnagar, Nepal. The study population included all patients presenting at the center during this period. Patients were eligible for inclusion if they presented with symptoms suggestive of tuberculosis, were new smear-negative cases with high clinical suspicion, were re-treatment cases, were people living with HIV with suspected TB, or remained smear-positive after 3 months of standard treatment. Specimens deemed inappropriate, such as saliva submitted as sputum, were excluded. Prior to enrollment, the study’s objectives were explained to all participants, and verbal informed consent was obtained. All eligible consecutive TB suspects presenting to the DOTS-Plus Centre during the study duration were included, yielding 509 samples. Based on the observed TB positivity rate of 31.2% in this data set (see Prevalence and comparative diagnostic yield), the 95% confidence interval (CI) corresponds to a precision of approximately ±4.1%. Therefore, the sample size was sufficient to provide reliable estimates of diagnostic performance and the prevalence of rifampicin resistance in this high-burden setting.

### Laboratory procedures

All clinical specimens were collected and processed at the NATA DOTS-Plus Centre, Biratnagar. For sputum samples, patients provided two specimens (one early morning and one spot) in sterile, leak-proof containers. Each specimen was vortexed thoroughly to achieve homogeneity and ensure even distribution of bacilli. From the homogenized sample, 1 mL aliquots were taken for smear microscopy and Xpert MTB/RIF testing to minimize sampling bias between diagnostic methods.

For acid-fast bacilli microscopy, smears were prepared directly from homogenized sputum, air-dried, heat-fixed, and stained by both ZN and auramine-O fluorescence methods using standard protocols (22). ZN-stained smears were examined under oil immersion (1,000×) using a bright-field microscope, and fluorescent-stained smears were examined at 400× using an LED fluorescence microscope.

For Xpert MTB/RIF testing, 1 mL of homogenized sputum was mixed with 2 mL of Xpert sample reagent in the collection tube. The mixture was vortexed for 10 s, incubated at room temperature for 10 min, re-vortexed, and finally incubated for an additional 5 min. Two milliliters of the processed mixture was then transferred into the Xpert MTB/RIF cartridge, and the test was performed following the manufacturer’s instructions (Cepheid, Sunnyvale, CA, USA).

#### Biosafety

All laboratory procedures were performed in a certified Class II biological safety cabinet in a BSL-3 facility. Laboratory personnel wore N95 respirators, gloves, eye protection, and disposable laboratory gowns. Work surfaces were decontaminated with 0.5% sodium hypochlorite after each procedure, and infectious waste was autoclaved prior to disposal. These practices conform with WHO and national tuberculosis laboratory biosafety recommendations.

### Data collection and statistical analysis

Demographic and clinical variables, including HIV status, smear microscopy results, and Xpert MTB/RIF outcomes, were extracted from the routinely maintained IOM TB REACH Project logbook. Data were coded and analyzed using IBM SPSS Statistics for Windows, version 20.0 (IBM Corp., Armonk, NY, USA). Descriptive statistics summarized participant characteristics and diagnostic yields. Associations between categorical variables were assessed with the chi-square test, and Fisher’s exact test was used when expected cell counts were small. Odds ratios (ORs) with 95% confidence intervals quantified the strength of associations.

Because microscopy and Xpert assays were performed on matched specimens, McNemar’s test was used for paired comparisons of sensitivities, and Cohen’s kappa coefficient was used to assess agreement between tests. Exact (Clopper–Pearson) 95% CIs were calculated for sensitivity, specificity, and prevalence estimates. A two-sided *P*-value <0.05 was considered statistically significant.

Xpert results reported as “Error” or “No Result” were documented and repeated once if a new specimen was available. Unresolved errors were excluded from paired diagnostic analyses. For diagnostic accuracy calculations (sensitivity and specificity), the Xpert MTB/RIF assay was used as the operational reference standard because culture was not available for routine programmatic use, and Xpert results inform treatment decisions in the national program. To avoid misleading estimates from heterogeneous, low-count specimen types, paired diagnostic accuracy metrics were restricted to sputum specimens with valid paired results (*n* = 446). Extrapulmonary specimen results are presented separately in Table 2 and discussed qualitatively.

## RESULTS

### Study population and sample distribution

A total of 509 patients with suspected tuberculosis were enrolled in the study, from whom 509 clinical specimens were collected. Sputum was the predominant specimen, accounting for 87.6% (*n* = 446) of the total samples. The remaining 12.4% (*n* = 63) consisted of various extrapulmonary specimens, with bronchoalveolar lavage (BAL) (*n* = 20), gastric lavage (GL) (*n* = 16), and pus (*n* = 13) being the most frequent. A detailed breakdown of all collected sample types is provided in Table 1.

**TABLE 1:** Distribution of clinical samples (*N* = 509)

Sample type	Number of samples (*n*)	Percentage (%)
Sputum	446	87.6
BAL	20	3.9
GL	16	3.1
Pus	13	2.6
Pericardial fluid	9	1.8
Cerebrospinal fluid	1	0.2
Urine	1	0.2
Fine needle aspiration	1	0.2
Ascitic fluid	1	0.2
Synovial fluid	1	0.2
Total	509	100.0

The study population consisted of 363 males (71.3%) and 146 females (28.7%). Participants’ ages ranged from under 15 to over 56 years, with the largest cohort (*n* = 198, 38.9%) being 56 years of age or older. No statistically significant association was observed between the prevalence of TB and the patients’ gender (*P* = 0.157) or age group (*P* = 0.227).

### Prevalence and comparative diagnostic yield

Of the 509 samples analyzed, the overall prevalence of *Mycobacterium tuberculosis* as determined by the Xpert MTB/RIF assay was 31.2% (159 positive cases). The Xpert MTB/RIF assay demonstrated a significantly higher diagnostic yield compared to microscopy methods. It identified 159 positive cases, whereas FM detected 109 cases, and ZN staining detected 89 cases. This corresponds to a 78.7% increase in detected positives over ZN staining and a 45.9% increase over FM. In turn, FM also showed a higher diagnostic yield than the conventional ZN staining method.

### Test performance by sample type

The performance of each diagnostic test varied considerably by sample type, as detailed in Table 2. Among the 446 sputum samples, GeneXpert identified 145 positive cases, FM detected 102, and ZN detected 87. For extrapulmonary samples, GeneXpert was the most effective method, identifying MTB in BAL, pericardial fluid, and pus samples where microscopy methods detected fewer or no positive cases. Notably, no MTB was detected by any method in several single extrapulmonary samples (cerebrospinal fluid [CSF], urine, ascitic fluid, and synovial fluid).

**TABLE 2:** Distribution of test results by sample type

Sample type	Total (*n*)	ZN pos	ZN neg	FM pos	FM neg	Xpert pos	Xpert neg
Sputum	446	87	359	102	344	145	301
BAL	20	0	20	3	17	6	14
Pericardial fluid	9	0	9	0	9	2	7
Pus	13	2	11	3	10	6	7
Gastric lavage	16	0	16	0	16	0	16
Fine needle aspiration	1	0	1	1	0	0	1
Other^*a*^	4	0	4	0	4	0	4
Total	509	89	420	109	400	159	350

^*a*^ Others include CSF, urine, ascitic fluid, and synovial fluid (*n *= 1 each).

### Diagnostic accuracy of microscopy methods

In sputum-only paired analyses (*n* = 446; sputum Xpert-positive = 145), FM demonstrated higher sensitivity than ZN staining: 70.3% (102/145; 95% CI, 62.2–77.6) vs 60.0% (87/145; 95% CI, 51.5–68.0). Specificity for both microscopy methods against Xpert was 100.0% (301/301; 95% CI, 98.8–100.0). The difference in paired sensitivities was statistically significant (McNemar’s test, *P* < 0.001) (Table 3).

**TABLE 3:** Diagnostic accuracy of microscopy methods^*a*^

Comparison	Sensitivity (%)	95% CI	Specificity (%)	95% CI
ZN staining vs Xpert	60.0	51.5–68.0	100.0	98.8–100.0
Fluorescence vs Xpert	70.3	62.2–77.6	100.0	98.8–100.0
ZN staining vs FM	85.3	76.9–91.5	100.0	98.8–100.0

^*a*^ Paired diagnostic accuracy calculations were restricted to sputum specimens (*n* = 446; sputum Xpert positives = 145). Extrapulmonary specimens were excluded from paired analyses due to low numbers and specimen heterogeneity.

### Rifampicin resistance and relapse cases

Among the 159 MTB-positive cases detected by GeneXpert, rifampicin resistance was found in 8/159 Xpert positives (5.03%; 95% CI: 2.2%–9.7%). Additionally, nine cases (5.7%) were identified as infections in previously treated patients. During the study, four tests (0.8% of total assays) resulted in an “Error” from the GeneXpert instrument, which were attributed to power interruptions. The prevalence of resistance was significantly associated with patient treatment history (*P* < 0.001, Fisher’s exact test). Of the eight resistant cases, six (75%) occurred in previously treated patients, whereas only two (25%) were identified in new patients. The rate of rifampicin resistance was 66.7% (6/9) among previously treated patients, compared to just 1.3% (2/150) among new cases (Table 4). The odds of rifampicin resistance were approximately 148 times higher in previously treated patients compared with new cases (OR 148.0; 95% CI, 20.7–1,057.7; *P* < 0.001, Fisher’s exact test).

**TABLE 4:** Rifampicin resistance by patient treatment history

Patient history	Rifampicin-resistant, *n* (%)	Rifampicin-sensitive, *n* (%)	Total, *n* (%)	Odds ratio (95% CI)	*P*-value
Previously treated	6 (66.7)	3 (33.3)	9 (100)	148.0 (95% CI: 20.7–1,057.7)	<0.001^*a*^
New case	2 (1.3)	148 (98.7)	150 (100)		
Total	8 (5.0)	151 (95.0)	159 (100)		

^*a*^ Fisher’s exact test.

## DISCUSSION

The results of this study, conducted in Biratnagar, reveal the alarming public health issue of drug-resistant tuberculosis. A significant finding is the detection of rifampicin resistance in 5.03% of MTB-positive cases. This finding carries significant implications for Nepal’s National Tuberculosis Program, as RR-TB represents the hallmark of multidrug-resistant tuberculosis. The observed prevalence is higher than the national average of 2.2% in new cases, but lower than the 10.1% among previously treated patients (9).

The study population’s demographic profile demonstrates a common disease distribution for tuberculosis. There was a marked male predominance (71.3%) and a considerable proportion of older adults (38.9% aged 56 and above), aligning with the tuberculosis epidemiology observed in many high-burden countries, including Nepal (23). The predominance of sputum samples (87.6%) is anticipated, as pulmonary TB is the most prevalent and easily transmissible form of the disease (24). The inclusion of extrapulmonary specimens, though smaller in proportion (12.4%), reflects an important diagnostic approach, as TB can present in various forms that require targeted management. One of the most important observations is the absence of a statistically significant association between the TB disease burden and the patient’s gender or age. This indicates that among this group of clinically presumed cases, the initial clinical judgment or suspicion that triggered testing was a stronger influence than demographic information, reinforcing the notion that all patients with pertinent signs and symptoms must be evaluated, irrespective of age or gender (25).

The management of MDR-TB is extremely challenging due to the prolonged duration, high cost, and severe side effects of the treatment (10, 26). Each delayed or misdiagnosed case allows resistant strains to spread within the population, perpetuating transmission, increasing treatment failure, and incurring greater healthcare costs (27). It presents a formidable challenge in a country like Nepal, where there is limited routine surveillance due to culture and uneven access to diagnostics (20). The stealthy spread of MDR-TB hinders national control efforts of TB elimination.

The extremely high odds ratio for rifampicin resistance observed among previously treated patients (OR 148.0, 95% CI: 20.7–1,057.7) (Table 4) likely reflects multiple interacting causes. Patient-level drivers can include poor adherence or interrupted therapy due to socioeconomic barriers or adverse effects; system-level failures may include intermittent drug supply or inadequate treatment monitoring that allows resistant strains to be selected (28). Microbiologically, resistance can arise via selection of a minor resistant subpopulation during inadequate therapy or via reinfection with a resistant strain in the community. Given the small number of previously treated cases in our sample (*n* = 9), the estimate is imprecise but still indicates a pronounced programmatic problem. These findings support targeted policies such as routine rapid molecular (Xpert) testing for all retreatment cases and the need for molecular epidemiology to differentiate relapse from reinfection (29, 30).

The Xpert MTB/RIF assay simultaneously detects TB and rifampicin resistance within 2 h, replacing the weeks or months of delay associated with culture-based methods. This rapid turnaround enables clinicians to promptly initiate second-line therapy, reducing diagnostic delays and improving patient outcomes. This contributes to controlling MDR-TB transmission by enabling early intervention through the prompt initiation of appropriate treatment (31). It is particularly valuable as it helps prevent the amplification of further drug resistance and is economically prudent by reducing the need for costly hospitalizations (32, 33).

Beyond its role in combating drug resistance, the Xpert assay demonstrated a vastly superior ability to detect TB in general, addressing Nepal’s profound case detection gap. With a national treatment coverage of only 54%, nearly half of all TB cases are “missing” from the health system (9). Our study provides strong evidence that these cases can be found. The Xpert assay identified a TB prevalence of 31.2%, representing a 78.7% and 45.9% increase in bacteriologically confirmed cases over ZN and FM, respectively. This superior yield is due to its ability to detect small amounts of *M. tuberculosis* DNA in paucibacillary samples, those with low bacterial loads that are systematically missed by microscopy, which requires a high concentration of bacilli for visual detection (34).

Xpert’s molecular advantages were especially pronounced for certain extrapulmonary (EPTB) samples, such as bronchoalveolar lavage and pericardial fluid. Xpert was able to identify cases that were missed entirely with microscopy. Given that EPTB is notoriously difficult to diagnose due to the paucibacillary nature of specimens, the use of Xpert for these cases is critical for reducing the significant morbidity and mortality associated with delayed EPTB diagnosis (35). Another critical advantage of Xpert over microscopy lies in its specificity for MTBC. While microscopy cannot distinguish MTBC from NTM, Xpert’s molecular detection prevents misclassification and inappropriate anti-TB therapy (36). This is clinically significant given the rising prevalence of NTM-related pulmonary disease globally, including across Asia, where clinical presentations are often indistinguishable from TB (15). This capability is the key to finding the “missing” cases and is strongly supported by the WHO’s recommendation to use Xpert as the initial diagnostic test for all adults and children with signs and symptoms of pulmonary and certain forms of EPTB (37).

However, the Xpert assay’s accuracy and efficiency do not result in a public health effectiveness without significant operational and financial considerations. The 0.8% error rate due to power interruptions in our study is a snapshot of the more systemic challenges present in many lower- and middle-income countries (38, 39). All of the operational issues work in conjunction to eliminate the primary advantage of speed and result in a loss of confidence in the entire system. Additionally, the higher per-test cost compared to microscopy remains a barrier. While the financial analysis shows the savings and better outcomes, multiple trials have demonstrated that these benefits are only realized when the entire clinical pathway is optimized (32).

This study has several limitations. First, the use of the Xpert MTB/RIF assay as the reference standard, instead of the traditional “gold standard” of mycobacterial culture, was a pragmatic choice reflecting the clinical realities in our setting where culture is not available for rapid diagnosis. This approach measures the operational utility of microscopy against the best available rapid test but may not reflect its performance against culture. Second, because culture and species identification were not performed, we cannot exclude that a subset of smear-positive specimens represented NTM. This limitation may have inflated microscopy false-positive rates relative to culture and should temper interpretation of specificity estimates. Third, the Xpert assay detects DNA and cannot distinguish between live and dead bacilli, making it unsuitable for monitoring treatment response. Finally, its resistance detection is limited to rifampicin, potentially missing isoniazid mono-resistance, which is also clinically significant.

This investigation offers valuable insight confirming the substantial benefits of molecular diagnostics when compared to traditional microscopy in a high-burden context. We detected a high burden of tuberculosis (31.2%) along with a concerning rate of rifampicin resistance (5.03%) in the region of Biratnagar, which is considerably higher than the national average for new cases. The Xpert MTB/RIF assay proved to be invaluable in not only increasing the overall case detection by >30% compared to the best microscopy but also in the accurate diagnosis of TB in some paucibacillary extrapulmonary specimens.

Thus, the national focus should be on the epidemiological strategic expansion and enhanced utilization of the GeneXpert tool. While cost and infrastructure pose serious operational hurdles, they do not undermine the promise of the technology. Addressing the inadequacy of the national diagnosis gap using molecular diagnostics, providing a consistent supply of cartridges, and training the required personnel are the basic steps that need to be undertaken in order to reduce the transmission of MDR-TB and progress toward the TB elimination goal. Furthermore, the integration of next-generation molecular assays such as Xpert MTB/RIF Ultra, endorsed by the WHO and characterized by a lower limit of detection, has the potential to substantially enhance the identification of paucibacillary and extrapulmonary TB cases. Comparative evaluation of the Ultra assay against Xpert MTB/RIF represents an important priority for future diagnostic research in Nepal.

## Data Availability

Anonymized data set supporting this study is available from the corresponding author upon reasonable request and following approval by the Nepal Anti-Tuberculosis Association.
